# Passive radiant cooling without sacrificing the aesthetics of objects

**DOI:** 10.1038/s41377-022-00895-5

**Published:** 2022-06-27

**Authors:** Przemysław Jacek Dereń

**Affiliations:** Institute of Low Temperature and Structure Research PAS, ul. Okólna 2, Wrocław, Poland

**Keywords:** Optics and photonics, Physics, Optical materials and structures

## Abstract

Photonic-engineered passive radiative cooling built into decorative climate-controlled enclosures reduces the active power consumption of the existing enclosure without sacrificing its aesthetics.

## Abstract (web summary)

The shield and black wire symbolize two layers necessary for passive radiative cooling. The first protects from solar radiation, the second connects to Space functioning as a heat sink.

## Graphical abstract

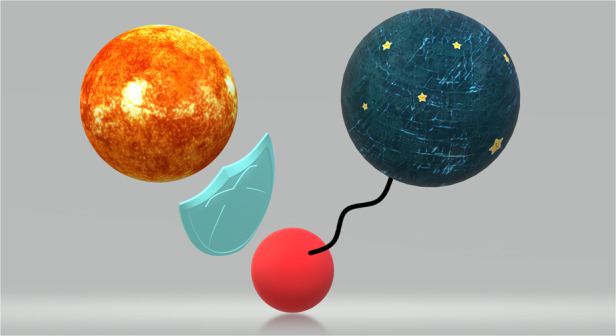

Faced with the rise in the Earth’s temperature and climate change making our planet hotter and with the growing demand for energy for air conditioning, it is necessary to limit ourselves and turn to solutions that have been used for centuries to cool our homes, however, by adding our modern knowledge of physics and materials engineering.

Passive cooling has been known for hundreds of years. By analyzing the structures of houses built in the Roman Empire, we can see how the architecture helped people survive heatwaves and live in a hot climate. The Sun radiation was not allowed to enter the interior of the house, placing it suitably along with the cardinal directions. White paints reflected of solar radiation up to 85%, avoiding significant heating of the walls^1^. Appropriately shaped roofs shielded the interior of the atrium, and the pool at its center helped reduce the temperature inside the house through evaporation.

In ancient times, in the Middle East, despite the lack of knowledge on the physical basis of the phenomenon, the wind lower the temperature inside the building by adiabatic cooling, and passive radiative cooling found a practical application. During cloudless nights in the dry climate, passive radiative cooling was employed to produce ice^2^.

To understand the mechanism of such passive radiative cooling, and then use it to lower the temperature inside the house, it was necessary to determine the transmission windows of the Earth’s atmosphere for electromagnetic waves^3^, to describe the spectral density of electromagnetic radiation emitted by a blackbody^4^, and to develop photonics for the preparation of interference filters or even two-dimensional photonic crystals with the appropriate emissivity^5,6^.

A blackbody with a temperature of 300–330 K radiates its energy with a maximum in the transmission window of the atmosphere in the range of 8–13 micrometers^7,8^. This way, during cloudless nights, heat can be radiated directly into Space which works as a huge heat sink. However, to improve the efficiency of cooling, it was necessary to “attach” a cooled surface to the Space by applying a high-emissivity material in the atmospheric window. Already in the mid-twentieth century, it was noticed that this property of the atmosphere could allow passive radiative cooling of an object even in sunny weather, assuming that its surface is protected by a solar reflective layer and has high emissivity properties within 8–13 µm^9,10^. Recently, the practical application of passive cooling is presented in the works of A.P. Raman et al.^5^. They achieved a cooling efficiency of 40 W/m^2^ from a surface directly illuminated by sunlight whose power density exceeds 850 W/m^2^.

The work of Zhu et al.^11^ recently published in *Light: Science & Applications* has an important impact on passive radiative cooling research. However, the authors were primarily interested in showing how passive cooling can be integrated into existing active refrigeration systems. They also point out that existing objects with cooled interiors have their own finishes that have aesthetic properties such as vehicle colors. Therefore, they decided to study how to implement passive cooling on decorative casings of such objects. The main goal of the research was to reduce the energy consumption to cool the existing temperature-controlled housing without sacrificing its aesthetics. This system has been called Enhanced Color-preserving Radiative Cooling (ECRC).

They prepared an ECRC outdoor cooling experiment for active temperature-controlled enclosures. The exterior and interior sides of the roof of the housing were covered with films transparent to visible light. The exterior consists of a SiO_2_/TiO_2_ stack, which transmitted about 80% of the visible part of the solar spectrum only, i.e., in the range of 400–800 nm, and reflected the UV and infrared part of the solar radiation, while the 6–14 micrometer zone is characterized by a high emissivity of 90%. The second inner ITO/PET layer was a barrier to heat transmission, but it did transmit visible light. In addition to ECTC layers, commercial hot mirrors were tested in this experiment.

Indoor temperature and heat flow tests were performed for three enclosures: with bare roofs, ECRC, and hot mirrors, as well as at three stages: when the active cooler is off, for constant power consumption, and at constant room temperature. When the cooler was turned off, the application of the hot mirrors and ECRC slightly lowered the temperature in the room almost in the same way. At the stage of constant input power to the active cooling, the internal temperature of the ECRC was 9.6 °C lower than that of the bare roof enclosure. On the other hand, the third stage measurements showed that the use of ECRC leads to energy savings of up to 63%. In addition, the performance of this system has been tested for four different roof colors. In all cases, the lowest indoor temperature was for ECRC. However, the roof temperature with ECRC was still higher than for the hot mirror due to an additional inner layer and thus obstructing the inner radiation channel and increasing outward radiant cooling of the enclosure as a whole.

The results presented in this work are important because they show that at a really low cost, it is possible through passive radiative cooling to significantly reduce the energy consumption for air conditioning, for example, of vehicles and, above all, the use of this technique will not alter the aesthetics of the objects.
